# Effect of intra-partum Oxytocin on neonatal encephalopathy: a systematic review and meta-analysis

**DOI:** 10.1186/s12884-021-04216-3

**Published:** 2021-10-30

**Authors:** Constance Burgod, Stuti Pant, Maria Moreno Morales, Paolo Montaldo, Phoebe Ivain, Ramyia Elangovan, Paul Bassett, Sudhin Thayyil

**Affiliations:** 1 Centre for Perinatal Neuroscience, Imperial College London, Du Cane Road, London, W12 0HS UK; 2 Neonatal Unit, Università degli Studi della Campania “Luigi Vanvitelli”, Naples, Italy; 3 Statsconsultancy Ltd., Amersham, London, England

**Keywords:** Oxytocin, Labour, Neonatal encephalopathy, Induction, Augmentation, Low- and middle-income countries, High-income countries

## Abstract

**Background:**

Oxytocin is widely used for induction and augmentation of labour, particularly in low- and middle-income countries (LMICs). In this systematic review and meta-analysis, we examined the effect of intra-partum Oxytocin use on neonatal encephalopathy.

**Methods:**

The protocol for this study was registered with PROSPERO (ID: CRD42020165049). We searched Medline, Embase and Web of Science Core Collection databases for papers published between January 1970 and May 2021. We considered all studies involving term and near-term (≥36 weeks’ gestation) primigravidae and multiparous women. We included all randomised, quasi-randomised clinical trials, retrospective studies and non-randomised prospective studies reporting intra-partum Oxytocin administration for induction and/or augmentation of labour. Our primary outcome was neonatal encephalopathy. Risk of bias was assessed in non-randomised studies using the Risk Of Bias In Non-randomised Studies of Interventions (ROBINS-I) tool. The RoB 2.0 tool was used for randomised studies. A Mantel-Haenszel statistical method and random effects analysis model were used for meta-analysis. Odds ratios were used to determine effect measure and reported with 95% confidence intervals.

**Results:**

We included data from seven studies (6 Case-control studies, 1 cluster-randomised trial) of which 3 took place in high-income countries (HICs) and 4 in LMICs. The pooled data included a total of 24,208 women giving birth at or after 36 weeks; 7642 had intra-partum Oxytocin for induction and/or augmentation of labour, and 16,566 did not receive intra-partum Oxytocin. Oxytocin use was associated with an increased prevalence of neonatal encephalopathy (Odds Ratio 2.19, 95% CI 1.58 to 3.04; *p* < 0.00001).

**Conclusions:**

Intra-partum Oxytocin may increase the risk of neonatal encephalopathy. Future clinical trials of uterotonics should include neonatal encephalopathy as a key outcome.

**Supplementary Information:**

The online version contains supplementary material available at 10.1186/s12884-021-04216-3.

## Background

Clinical administration of exogenous Oxytocin is the most common way to induce and augment labour as it increases the frequency, duration and strength of uterine contractions [1]. Recent data suggest that intra-partum Oxytocin is used in 18.6 to 51% [2, 3] of institutionalised deliveries, both in high-income countries (HICs) and in low- and middle-income countries (LMICs) [4].

Intra-partum Oxytocin administration has been shown to reduce the mean duration in labour in several clinical trials [5]. This is a major benefit as a prolonged labour is often correlated with a higher risk of post-partum haemorrhage and maternal death, particularly in LMICs [6]. Nevertheless, rates of caesarean section and instrumental deliveries were not significantly different in women administered with Oxytocin compared to no treatment [5]. Furthermore, early administration of Oxytocin has been associated with uterine hyperstimulation and fetal heart rate changes, which may put the foetus at risk of a reduction in oxygenation of the fetal brain. A Cochrane review comparing high doses and low doses of Oxytocin [7] reported no significant difference in caesarean section rates, vaginal delivery not achieved within 24 h or neonatal morbidity/perinatal death (such as seizures, birth asphyxia, neonatal encephalopathy and disability in childhood).

Improper use and administration of high doses of Oxytocin has been found to precede perinatal sentinel events such as uterine rupture, cord prolapse or placental abruption as a result of uterine hyperstimulation, leading to fetal asphyxia [8]. Inappropriate use of Oxytocin may be particularly common in LMICs. Recent data suggest that the fetal brain injury often occurs without acute perinatal sentinel events in these settings and may be unresponsive to conventional therapies like therapeutic hypothermia [9]. Hence, we wanted to assess the effect of intra-partum Oxytocin for labour induction/augmentation on neonatal encephalopathy.

## Methods

### Data source

The protocol for this study was registered with PROSPERO (ID: CRD42020165049). Any amendment or modification to the original protocol during the course of the review was submitted to PROSPERO. The Cochrane handbook for systematic reviews of interventions was used to frame this review. One investigator (CB) searched the published literature between January 1970 to May 2021 on Medline (Ovid), Embase (Ovid) and Web of Science Core Collection (Web of Science, all citation indexes) databases to access relevant studies. The years were limited to 1970 to reflect current clinical practice as much as possible to allow the translation of findings. We searched for the concepts (1) encephalopathy/HIE, (2) oxytocin/uterotonic, expanded with risk factors and intrapartum injections/treatment/intervention/management/surveillance to find papers not explicitly mentioning oxytocin and (3) newborn infants/newborn infant diseases and obstetric, using controlled terms (i.e. MeSH-terms in MEDLINE) and words in title and abstract. (Supplementary Table 1). All languages of origin were accepted provided the papers could be translated to English. Bibliographies and reference lists of the selected articles were also examined to collate any further relevant references not picked up by the initial search. Full text of articles identified by the search and considered to meet the inclusion criteria, based on their title, abstract and subject descriptors were obtained for data synthesis. All search results were combined into a single database of references in EndNote (version X9.3.3). Additional papers citing any of the relevant studies selected following full text screening were searched using Google Scholar.

### Study selection

We considered all randomised controlled trials (RCTs), quasi RCTs, non-randomised prospective cohort studies and retrospective studies reporting intrapartum use of Oxytocin.

#### Population

Primigravidae and multiparous women giving birth at or after 36 weeks’ gestation.

#### Intervention

Intra-partum Oxytocin for induction (continuous and discontinuous) and augmentation of labour through intravenous and intramuscular routes at all doses. We excluded studies where Oxytocin was used after the delivery of the baby for prevention of post-partum bleed.

#### Comparison

Women who did not receive Oxytocin or any other uterotonic drug during labour.

#### Outcome

Babies with signs of neonatal encephalopathy following Oxytocin administration during labour. Neonatal encephalopathy was defined as the need for prolonged resuscitation at 5 min of age and/or a 5-min Apgar score < 7 (for babies born in hospital) or lack of crying by 5 min of age for babies born at home.

### Data extraction and analysis

Raw data was extracted from the selected studies on Excel spreadsheets by two independent investigators (CB/MM) and cross-checked by a third investigator (PM). Any disagreement during study selection or data extraction and analysis were resolved by consensus or by involving a senior reviewer when no consensus was reached. Risk of bias was assessed for each study by one investigator (CB) using the Risk Of Bias In Non-randomised Studies of Interventions (ROBINS-I) tool, which evaluates risk of bias in estimates of the effectiveness or safety (benefit or harm) of an intervention. The RoB 2.0 tool was used to assess the risk of bias of randomised studies. Grading of Recommendations, Assessment, Development and Evaluations (GRADE) was used to assess certainty in the body of evidence. A Mantel-Haenszel statistical method and random effects analysis model were used for meta-analysis. Odds ratios were used to determine effect measure and reported with 95% confidence intervals. Heterogeneity between studies was assessed to determine variability in the data using the I^2^ index. An I^2^ value of size 0–40% was considered a low, 30–60% as moderate, 50–90% as substantial and 75–100% as considerable heterogeneity according to the Cochrane Handbook [10]. Subgroup effects measure according to settings (HICs and LMICs) were calculated using odds ratios, and heterogeneity within subgroups was also measured using the I^2^ index. A Chi^2^ test was conducted to determine subgroup differences. Lastly, the overall effect was presented using a Z-test. All statistical analyses were performed on the RevMan V.5.1.4 software.

## Results

**Fig. 1:**
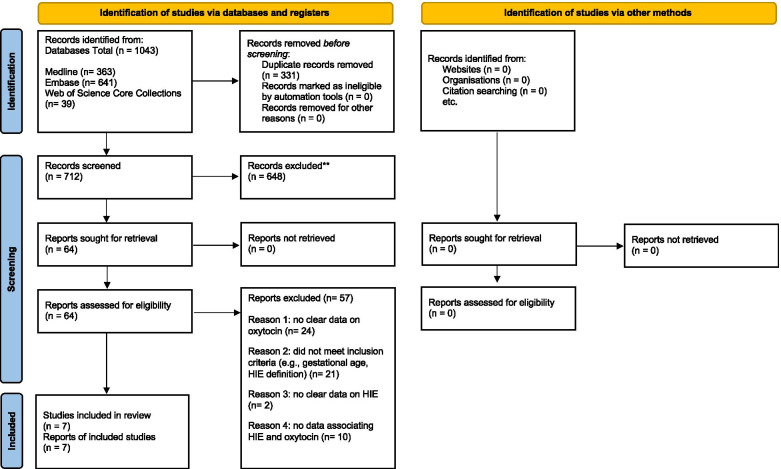
Flow chart of the literature search

We identified seven studies following full text screening, of which one had its data collected during a previous community-based cluster randomised controlled trial. These seven studies included neonatal encephalopathy as an outcome, of which 6 were case controls and 1 was a cluster randomised trial [11–17] (Fig. 1). Overall, 101 studies were excluded after full-text screening. Sixty-two studies were excluded as they did not have clear data on Oxytocin, 20 were excluded as they did not meet inclusion criteria such as gestational age or the definition of neonatal encephalopathy, 11 further studies did not have clear data on neonatal encephalopathy and lastly, eight studies were excluded due to the inability to extract data reflecting Oxytocin use in neonatal encephalopathy babies.

Inclusion criteria for participants in selected studies were comparable: (1) a gestational age of at least 36 weeks with (2) a classification of the baby into mild, moderate or severe neonatal encephalopathy according to Sarnat scoring and (3) administration of Oxytocin for induction and/or augmentation of labour. Studies compared neonatal encephalopathy infants with term infants with no sign of encephalopathy at birth (*n* = 4 studies), asphyxiated infants (Apgar score of < 6 at 1 min) with no sign of encephalopathy (*n* = 1 study) or term infants with a Thompson score < 3 (*n* = 1 study). Data from the remaining study compared women with no injections during labour and women with injections during labour. Three of the included studies were undertaken in HICs, 4 in LMIC settings and all reported Oxytocin use and the prevalence of neonatal encephalopathy in newborns (Supplementary Table 2).

Two of the studies, however, did not use Sarnat scoring to determine neonatal encephalopathy severity*. Ellis* et al [14] assessed encephalopathy severity according to criteria derived from the syndromic descriptions of Fenichel modified. *Tann et al* [17] employed the Thompson score to examine for encephalopathy, severity was then graded per modified Sarnat classification. *Mullany et al* [16] considered infants to have intrapartum-related moderate-severe neonatal encephalopathy provided they experienced intrapartum-related neonatal respiratory depression resulting in death of seizures and 2 of the following: lethargy, poor suck or respiratory rate less than 40 breaths per minute, observed anytime during the first 7 days of life. Lastly, *Farquhar* [11] *et al* describe the study to include only moderate and severe encephalopathic babies according to New Zealand’s Neonatal Encephalopathy Working Group of the Perinatal and Maternal Mortality Review Committee (PMMRC). The Committee defines neonatal encephalopathy as a clinically defined syndrome of disturbed neurological function within the first week of life in term infants (i.e., 37 weeks or older), manifested by difficulty initiating and maintaining respiration, depression of tone and reflexes, subnormal level of consciousness, and often, seizures. Upon further reading, we established that the PMMRC defines moderate and severe neonatal encephalopathy as Sarnat stages 2 and 3 [18].

Risk of bias was evaluated for 6 studies using the ROBINS-I tool, which is used in observational studies comparing health effects of interventions. The RoB 2.0 tool was used to assess for of bias of the cluster-randomised study. Selected studies ranged from low to severe risk of bias due to confounding domain and co-interventions, as these were not assessing specifically the effect of Oxytocin on neonatal outcomes (Supplementary Table 3, Supplementary Table 4).

**Fig. 2:**
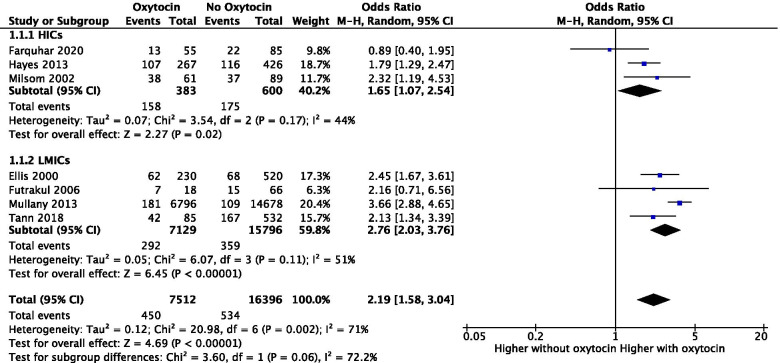
Forest plot of the effect of intra-partum Oxytocin administration on the onset of neonatal encephalopathy in HICs and LMICs. HICs, high income countries; LMICs; low- and middle-income countries

Pooled data from HIC studies (*Farquhar* et al, *Hayes* et al and *Milsom* et al) [11–13] revealed that 24, 40 and 62% of mothers that were administered Oxytocin gave birth to an encephalopathic infant, respectively. Moreover, the four studies looking at LMICs (*Ellis* et al*, Futrakul* et al*, Mullany* et al*, Tann* et al) [14–17] reported the same event occurring in 27, 39, 3 and 49% of infants following intra-partum Oxytocin administration. Out of women that were not administered intra-partum Oxytocin, *Farquhar et al* [11]*, Hayes et al* [12] and *Milsom et al* [13] showed that only 26, 27 and 42% of infants developed neonatal encephalopathy, respectively. Furthermore*, Ellis et al* [14]*, Futrakul et al* [15]*, Mullany et al* [16] and *Tann et al* [17] reported that only 13, 23, 0.7 and 31% of infants developed neonatal encephalopathy in the intra-partum Oxytocin-free group (Fig. 2).

Primary outcome (all grades of neonatal encephalopathy) was available for 158 infants in the Oxytocin group and 175 infants in the Oxytocin-free group in high-income settings. Pooled data from HIC studies showed a significant difference in the subtotal for the onset of neonatal encephalopathy between the Oxytocin (158/383; 41%) and Oxytocin-free (175/600; 29%) groups (Odds Ratio 1.65, 95% CI 1.07 to 2.54; *p* = 0.02). Furthermore, the subtotal difference in the onset of neonatal encephalopathy between the Oxytocin (292/7129; 4%) and the Oxytocin-free (359/15796; 2%) groups was significantly different in studies from LMICs (Odds Ratio 2.76, 95% CI 2.03 to 3.76; *p* < 0.00001). The overall effect combining results from both HICs and LMICs demonstrates a significant difference between the Oxytocin (450/7512; 6%) and Oxytocin-free group (534/16396; 3%) in the onset of neonatal encephalopathy (Odds Ratio 2.19, 95% CI 1.58 to 3.04; *p* < 0.00001). Subgroup differences analysis (I^2^) revealed that 72.2% of the variability in effect estimates from both groups is due to subgroup differences rather than sampling error (Fig. 2).

The included studies had an overall substantial heterogeneity (I^2^ = 71%). The heterogeneity amongst subgroups was of 44% for HIC studies and 51% for LMIC studies.

## Discussion

We assessed a total of seven studies from HICs (*n* = 3, three case controls studies) and LMICs (*n* = 4, three case controls studies, one cluster-randomised trial) that included intra-partum Oxytocin use in mothers of term infants with neonatal encephalopathy. Our main finding was an association between Oxytocin use and the onset of neonatal encephalopathy, which was significantly higher in term babies of women induced/augmented with Oxytocin compared to Oxytocin-free women (OR 2.19, 95% CI 1.58 to 3.04; *p* < 0.00001). Moreover, this was found to be even higher in LMICs (OR 2.76, 95% CI 2.03 to 3.76; *p* < 0.00001) compared to HICs (OR 1.65, 95% CI 1.07 to 2.54; *p* = 0.02). A higher percentage of heterogeneity across LMIC studies may be linked to variability in social environment as well as settings where studies were taking place such as home births in rural areas of southern Nepal [16].

Although we acknowledge that systematic reviews of RCTs are more likely to provide unbiased information, we justify the inclusion of non-randomised studies in the presented review to be due to the extremely unlikeliness of this area of interest to be studied in randomised trials.

The manifestation of antepartum and intrapartum factors or events may foresee neonatal encephalopathy onset and severity [19]. Complications are often related to the dosage and duration of administration, with induction with higher doses of Oxytocin generally being associated with an escalation in uterine contractions, often leading to dangerous uterine hyperstimulation [8].

While there may be a link between injudicious Oxytocin use and neonatal encephalopathy, we need to emphasize that the presented studies were not looking specifically at the use of Oxytocin or uterotonics in women in labour. Therefore, we were not able to determine whether women receiving Oxytocin also experienced any perinatal sentinel events prior to delivering an encephalopathic infant. Hence the pooled Odds ratios were based on case control studies and a cluster-randomised trial and are prone to a multitude of confounding factors. Interestingly, most concluded that intra-partum Oxytocin administration for induction and/or augmentation was one of the main variables associated with an increased risk of neonatal encephalopathy.

Secondly, the method, precise indication, dose and frequency of Oxytocin administration was not available in most studies. As induction and augmentation were combined for meta-analysis, further research needs to be performed in this field to assess whether induction and/or augmentation with Oxytocin is of greater risk. For example, *Mullany et al* [16] specified that an important, if not large proportion of the injections reported comprised Oxytocin or other injectable drug with uterotonic effects. Injections may therefore have included Oxytocin or other injectable uterotonics, as well as antibiotics, vitamin B12, saline and antipyretics. Their study highlights the common practice of inappropriate use of uterotonics and other injectable drugs in LMIC settings, often by unqualified providers, and its relationship with poor outcomes.

Improper Oxytocin use is worrying, especially in LMIC settings, where the implementation of the partograph is suboptimal due to a lack of availability of labour management guidelines, insufficient training and supervision and a negative perception related to partograph use [20]. Labour induction guidelines in South Asia indicate that at-risk women should be excluded from intra-partum Oxytocin administration [21]. Additionally, solely nulliparous women should be eligible and only following artificial rupture of membranes in order to prevent amniotic fluid entering maternal circulation. Most importantly, progress of labour such as contractions and fetal heart rate should be monitored continuously, when possible, using a partograph and interventions should be recorded. Oxytocin should be immediately interrupted if hyperstimulation or fetal distress is identified.

Low-dose and high-dose regimens have been compared and although neonatal outcomes were not assessed, a higher occurrence of uterine hyperstimulation was reported with repeated high-dose regimens [22]. Furthermore, the National Institute for Health and Clinical Excellence (NICE) guidelines advise against Oxytocin use in the second stage of labour due to the heightened risk of uterine rupture [23]. However, Oxytocin for controlling post-partum haemorrhage in the third stage of labour must be maintained as it is effective in significantly reducing blood loss greater than 500 mL and maternal mortality [24].

## Conclusion

Our data suggest a potential association of intra-partum Oxytocin use with neonatal encephalopathy. The poor effectivity of intra-partum Oxytocin treatment in reducing rates of caesarean section and instrumental deliveries, as well as its association with uterine hyperstimulation and fetal heart rate changes, has already been shown in Cochrane systematic reviews [5, 7]. This should provide further confirmation and warn health professionals that intrapartum Oxytocin use may put newborns at risk of neonatal encephalopathy. Finally, it should lead to a review of clinical guidelines and improvement of intra-partum care, especially in LMICs, where the monitoring of fetal heart rate and uterine contractions needs to be emphasized, particularly alongside Oxytocin administration.

## Supplementary Information

**Additional file 1: Supplementary Table 1.** Search strategies for systematic review. **Supplementary Table 2.** Details of studies meeting the eligibility criteria. **Supplementary Table 3.** Risk of bias (ROBINS-I) summary. **Supplementary Table 4.** Risk of bias for Mullany et al., 2013 (RoB 2.0). **Supplementary Table 5.** Summary of Findings table (GRADE).

## Data Availability

All data generated or analysed during this study are included in this published article [and its supplementary information files]. The datasets used and analysed in the presented study are available online. No unpublished data was included.

## Abbreviations

HICs: High-Income Countries
LMICs: Low- and Middle-Income Countries
MESH: Medical Subject Headings
NICE: The National Institute for Health and Care Excellence
PMMRC: Perinatal and Maternal Mortality Review Committee
RCTs: Randomised Controlled Trials
ROBINS-I: Risk Of Bias In Non-randomised Studies - of Interventions
