# Supplementary material for: Effect of intra-partum Oxytocin on neonatal encephalopathy: a systematic review and meta-analysis
Source: BMC Pregnancy Childbirth. 2021 Oct 30;21:736. doi: 10.1186/s12884-021-04216-3 (PMC8556930; doi:10.1186/s12884-021-04216-3)
Supplement: Supplementary file 1 — Additional file 1: Supplementary Table 1. Search strategies for systematic review. Supplementary Table 2. Details of studies meeting the eligibility criteria. Supplementary Table 3. Risk of bias (ROBINS-I) summary. Supplementary Table 4. Risk of bias for Mullany et al., 2013 (RoB 2.0). Supplementary Table 5. Summary of Findings table (GRADE). [file 12884_2021_4216_MOESM1_ESM.docx]

**Supplementary Table 1: Search Strategies for systematic review**

Ovid MEDLINE(R) ALL <1946 to August 24, 2021>

1 Hypoxia-Ischemia, Brain/ 6151

2 hypoxic-ischem*.ti,ab,kf. 7385

3 hypoxic-ischaem*.ti,ab,kf. 1135

4 HIE*.ti,ab,kf. 114197

5 NHIE*.ti,ab,kf. 26

6 encephalopath*.ti,ab,kf. 53746

7 (hypox*-isch?em* or hypox*isch?em*).tw,kf. 10919

8 1 or 2 or 3 or 4 or 5 or 6 or 7 173989

9 exp Infant, Newborn/ 633224

10 exp Fetus/ 160968

11 exp Infant, Newborn, Diseases/ 180253

12 Asphyxia Neonatorum/ 7840

13 Fetal Hypoxia/ 3160

14 Perinatology/ 1858

15 (f?etus* or foetus* or f?etal or neonat* or neo-nat* or new*born* or new*-born* or postnat* or post-nat* or perinat* or peri-nat* or baby or babies).tw,kf. 844900

16 obstetric*.mp. 192696

17 exp Labor, Obstetric/ 47127

18 exp Delivery, Obstetric/ 84462

19 9 or 10 or 11 or 12 or 13 or 14 or 15 or 16 or 17 or 18 1429978

20 Oxytocin/ 20348

21 (oxytocin* or oxytocic* or uterotonic* or (uter* adj3 (stimula* or contract* or tonic*))).tw,kf. 32987

22 ((risk adj3 factor*) or (labo?r adj3 (augment* or induc*))).ti,kf. 165206

23 ((intrapart* or intra-part* or labo?r) adj3 (inject* or treat* or interven* or manag* or surveill*)).tw,kf. 6338

24 20 or 21 or 22 or 23 205270

25 8 and 19 and 24 363

26 limit 25 to yr="1970 -Current" 363

******************************************************************************************************

Embase Classic+Embase <1947 to 2021 August 24>

1 hypoxic ischemic encephalopathy/ 8581

2 hypoxic-ischem*.ti,ab,kw. 10457

3 hypoxic-ischaem*.ti,ab,kw. 1835

4 HIE*.ti,ab,kw. 132571

5 NHIE*.ti,ab,kw. 38

6 encephalopath*.ti,ab,kw. 84274

7 (hypox*-isch?em* or hypox*isch?em*).tw,kw. 15684

8 1 or 2 or 3 or 4 or 5 or 6 or 7 224827

9 exp newborn/ 643673

10 exp fetus/ 222751

11 exp newborn disease/ 1822239

12 newborn hypoxia/ 7042

13 fetus hypoxia/ 5053

14 perinatology/ 2429

15 (f?etus* or foetus* or f?etal or neonat* or neo-nat* or new*born* or new*-born* or postnat* or post-nat* or perinat* or peri-nat* or baby or babies).tw,kw. 1138761

16 obstetric*.mp. 207653

17 exp labor/ 43030

18 exp obstetric delivery/ 176273

19 9 or 10 or 11 or 12 or 13 or 14 or 15 or 16 or 17 or 18 3021533

20 oxytocin/ 38783

21 (oxytocin* or oxytocic* or uterotonic* or (uter* adj3 (stimula* or contract* or tonic*))).tw,kw. 43874

22 ((risk adj3 factor*) or (labo?r adj3 (augment* or induc*))).ti,kw. 250128

23 ((intrapart* or intra-part* or labo?r) adj3 (inject* or treat* or interven* or manag* or surveill*)).tw,kw. 8744

24 20 or 21 or 22 or 23 310257

25 8 and 19 and 24 645

26 limit 25 to yr="1970 -Current" 641

******************************************************************************************************

Web of Science Core Collection

**(TS=(hypoxic-ischem* OR hypoxic-ischaem* OR HIE* OR NHIE* OR encephalopath* OR (hypox*-isch?em* OR hypox*isch?em*))) AND (TS=(f?etus* OR foetus* OR f?etal OR neonat* OR neo-nat* OR new*born* OR new*-born* OR postnat* OR post-nat* OR perinat* OR peri-nat* OR baby OR babies OR obstetric*)) AND (((TS=(oxytocin* OR oxytocic* OR uterotonic* OR (uter* adh3 (stimula* or contract* or tonic*)))) OR (TI=((risk adhd factor*) OR (labo?r adhd (augment* OR induc*)))) OR (TS=((intrapart* OR intra-part* OR labo?r) adhd (inject* OR treat* OR interven* OR manag* OR surveill*)))))**

Timespan= 1970 – Current

Hits: 39

******************************************************************************************************

|  |  | | | | Supplementary Table 2. Details of Studies meeting the eligibility criteria | | |  |
| --- | --- | --- | --- | --- | --- | --- | --- | --- |
| Author and Year | **Country Income** | **Location and Date** | **Study Design** | **Total number of subjects** | | **Definition of controls in study** | **Evidence of neonatal encephalopathy** | **Oxytocin administration** |
| Farquhar et al., 2020 | High Income | New Zealand, 2010-2011 | Case-Control | Cases: 35  Controls: 105 | | Gestational age of 37 weeks or older with Apgar scores of 9 or 10 at 1 and 5 minutes, cord lactate of 4.8 mmol/L or less, at least 1 hour of CTG recordings from at least 2 hours before delivery, no evidence of NE, no congenital anomalies and no admission to a neonatal intensive care unit. | Infants without congenital anomalies who met the PMMRC definition of moderate to severe NE and showed evidence of hypoxia at birth. | Received oxytocin/uterotonics: 13 cases and 42 controls  Did not receive oxytocin/uterotonics: 22 cases and 63 controls |
| Hayes et al., 2013 | High income | Ireland, 2001-2008 | Case-control | Cases: 237  Controls: 489 | | Infants who were born before and after each case, provided they were born at 36 weeks of gestation or above, had no major congenital abnormality or any sign of encephalopathy in the neonatal period. | Infants who required admission to the NICU at 24 hours after delivery or less, with evidence of encephalopathy. | Received oxytocin/uterotonics: 107 cases and 160 controls  Did not receive oxytocin/uterotonics: 116 cases and 310 controls |
| Milsom et al., 2002 | High Income | Sweden, 1985-1991 | Case-control | Cases: 75  Controls: 75 | | The next infant born on the same delivery unit, of the same gender, at 37 weeks of gestation and with an Apgar score of 7 at 5 min. | Birth asphyxia defined as Apgar score <7 at 5min. Asphyxia related HIE subgroup classified into mild, moderate or severe according to criteria of Sarnat. | Received oxytocin/uterotonics: 38 cases and 23 controls  Did not receive oxytocin/uterotonics: 37 cases and 52 controls |
| Ellis et al., 2000 | Low Income | Nepal, 1995-1996 | Case-control | Cases: 131  Controls: 635 | | Infants with a gestational age greater than 37 weeks without evidence of neonatal encephalopathy. | Evidence of neurobehavioural disturbance a 6-24 hours after birth. Definition chosen to enable comparison with prevalence studies of hypoxic-ischaemic encephalopathy in other settings. All case infants had a 5 min Apgar score <7. | Received oxytocin/uterotonics: 62 cases and 168 controls  Did not receive oxytocin/uterotonics: 68 cases and 452 controls |
| Futrakul et al., 2006 | Low Income | Thailand, 1999-2000 | Case-control | Cases: 22  Controls: 62 | | Birth asphyxia defined by an Apgar score <6 at 1 min without a subsequent diagnosis of HIE. | Birth asphyxia defined by an Apgar score <6 at 1 min with subsequent HIE diagnosis based on the modified Sarnat score. | Received oxytocin/uterotonics: 7 cases and 11 controls  Did not receive oxytocin/uterotonics: 15 cases and 51 controls |
| Mullany et al., 2013 | Low Income | Nepal, 2002-2006 | Community-based cluster-randomized trial | Total number of live births in injection during labour group: 6796  Total number of live births in no injection during labour group: 14678 | | All other live births provided they did not have intrapartum-related moderate-severe neonatal encephalopathy. | Intrapartum related Neonatal Encephalopathy (IPR-NE) defined as intrapartum related neonatal respiratory depression (IPR-NRD) resulting in death/seizures 2 of: lethargy, poor suck, or respiratory rate <40 breaths per minute, observed anytime during the first 7 days after birth among full-term infants. | Received injections during labour: 181 babies developed IPR-NE  Did not receive injections during labour: 109 developed IPR-NE |
| Tann et al., 2018 | Low Income | Uganda, 2011-2012 | Unmatched case-control | Cases: 209  Controls: 408 | | Term infants with Thompson score <3 provided they did not have prior antibiotics administration, lived within 20km from the hospital and were in-born infants. | Thompson score >5 within 12 hours of birth. Graded mild, moderate, severe per modified Sarnat classification. | Received oxytocin/uterotonics: 42 cases and 43 controls  Did not receive oxytocin/uterotonics: 167 cases and 365 controls |

|  |  | |  |  | |  | | Supplementary Table 3. Risk of bias (ROBINS-I) summary | | | | |
| --- | --- | --- | --- | --- | --- | --- | --- | --- | --- | --- | --- | --- |
| Author Name and Year | **Bias due to Confounding** | **Bias in selection of participants into the study** | | | **Bias in classification of interventions** | | **Bias due to deviations from Intended interventions** | | **Bias due to missing data** | **Bias in measurement of outcomes** | **Bias in selection of the reported results** | **Overall Bias** |
| Farquhar et al., 2020 | At least one known important confounding domain, we expect serious residual confounding. | Selection into the study was related (but not very strongly) to intervention and outcomes and start of follow up and of intervention do not coincide. | | | Intervention status is not well defined | | Any deviations from intended interventions reflected usual practice. | | Data were reasonably complete. | The outcome measure was unlikely to be influenced by knowledge of the intervention received by study participants. | There is no indication of selection of the reported analysis from among multiple analyses. There is no indication of selection of the cohort or subgroup for analyses and reporting on the basis of the results. | Severe risk of bias |
| Hayes et al., 2013 | Confounding expected and reliability and validity of measurement of important domains were sufficient, no serious residual confounding expected. | Selection into the study may have been related to intervention and outcome and start of follow up and of intervention do not coincide for all participants. | | | Intervention status is well defined and some aspects of the assignments of intervention status were determined retrospectively. | | Any deviations from intended interventions reflected usual practice. | | Data were reasonably complete. | The outcome measure was unlikely to be influenced by knowledge of the intervention received by study participants. | There is no indication of selection of the reported analysis from among multiple analyses. There is no indication of selection of the cohort or subgroup for analyses and reporting on the basis of the results. | Moderate risk of bias |
| Milsom et al., 2002 | No confounding expected. | All eligible participants included, start of follow up and intervention coincided for each participant. | | | Intervention status is well defined and is based solely on information collected at the time of intervention. | | Any deviations from intended interventions reflected usual practice. | | Data were reasonably complete. | The outcome measure was unlikely to be influenced by knowledge of the intervention received by study participants. | There is no indication of selection of the reported analysis from among multiple analyses. There is no indication of selection of the cohort or subgroup for analyses and reporting on the basis of the results. | Low risk of bias |
| Ellis et al., 2000 | No confounding expected. | All eligible participants included, start of follow up and intervention coincided for each participant. | | | Intervention status is well defined is based solely on information collected at the time of intervention. | | Any deviations from intended interventions reflected usual practice. | | Data were reasonably complete. | The outcome measure was unlikely to be influenced by knowledge of the intervention received by study participants. | There is no indication of selection of the reported analysis from among multiple analyses. There is no indication of selection of the cohort or subgroup for analyses and reporting on the basis of the results. | Low risk of bias |
| Futrakul et al., 2006 | At least one known important confounding domain, we expect serious residual confounding. | Selection into the study was related (but not very strongly) to intervention and outcomes and start of follow up and of intervention do not coincide. | | | Intervention status is not well defined | | Any deviations from intended interventions reflected usual practice. | | Data were reasonably complete. | The outcome measure was unlikely to be influenced by knowledge of the intervention received by study participants. | There is no indication of selection of the reported analysis from among multiple analyses. There is no indication of selection of the cohort or subgroup for analyses and reporting on the basis of the results. | Serious risk of bias |
| Tann et al., 2018 | At least one known important confounding domain, we expect serious residual confounding. | Selection into the study was related (but not very strongly) to intervention and outcomes and start of follow up and of intervention do not coincide. | | | Intervention status is not well defined | | Any deviations from intended interventions reflected usual practice. | | Data were reasonably complete. The analysis addressed missing data and is likely to have removed any risk of bias. | The outcome measure was unlikely to be influenced by knowledge of the intervention received by study participants. | There is no indication of selection of the reported analysis from among multiple analyses. There is no indication of selection of the cohort or subgroup for analyses and reporting on the basis of the results. | Serious risk of bias |

**Supplementary Table 4: Risk of bias for Mullany et al., 2013 (RoB 2.0)**

| **Mullany et al., 2013** |  |
| --- | --- |
| Random sequence generation (selection bias) | + |
| Allocation concealment (selection bias) | + |
| Blinding of participants and personnel (performance bias) | - |
| Blinding of outcome assessment (detection bias) | - |
| Incomplete outcome data (attrition bias) | - |
| Selective reporting (reporting bias) | - |

**Supplementary Table 5: Summary of Findings table (GRADE).**

| **Summary of findings:** | | | | | | |
| --- | --- | --- | --- | --- | --- | --- |
| **Oxytocin compared to no oxytocin for neonatal encephalopathy** | | | | | | |
| **Patient or population**: neonatal encephalopathy  **Setting**:  **Intervention**: oxytocin  **Comparison**: no oxytocin | | | | | | |
| Outcomes | **Anticipated absolute effects^*^** (95% CI) | | Relative effect (95% CI) | № of participants  (studies) | Certainty of the evidence (GRADE) | Comments |
|  | **Risk with no oxytocin** | **Risk with oxytocin** |  |  |  |  |
| Neonatal Encephalopathy (overall) | 33 per 1 000 | **69 per 1 000** (51 to 93) | **OR 2.19** (1.58 to 3.04) | 23908 ( observational studies) | ⨁⨁⨁◯ MODERATE |  |
| Neonatal Encephalopathy (HICs) | 292 per 1 000 | **405 per 1 000** (306 to 511) | **OR 1.65** (1.07 to 2.54) | 983 ( observational studies) | ⨁⨁⨁◯ MODERATE |  |
| Neonatal Encephalopathy (LMICs) | 23 per 1 000 | **60 per 1 000** (45 to 80) | **OR 2.76** (2.03 to 3.76) | 22925 ( observational studies) | ⨁⨁⨁◯ MODERATE |  |
| ***The risk in the intervention group** (and its 95% confidence interval) is based on the assumed risk in the comparison group and the **relative effect** of the intervention (and its 95% CI).   **CI:** Confidence interval; **OR:** Odds ratio | | | | | | |
| **GRADE Working Group grades of evidence** **High certainty:** We are very confident that the true effect lies close to that of the estimate of the effect **Moderate certainty:** We are moderately confident in the effect estimate: The true effect is likely to be close to the estimate of the effect, but there is a possibility that it is substantially different **Low certainty:** Our confidence in the effect estimate is limited: The true effect may be substantially different from the estimate of the effect **Very low certainty:** We have very little confidence in the effect estimate: The true effect is likely to be substantially different from the estimate of effect | | | | | | |
